# Socioeconomic Status, Institutional Power, and Body Mass Index among Chinese Adults

**DOI:** 10.3390/ijerph182010620

**Published:** 2021-10-11

**Authors:** Weidong Li, Shuzhuo Li, Marcus W. Feldman

**Affiliations:** 1 Department of Sociology, School of Philosophy and Government, Shaanxi Normal University, Xi’an 710119, China; 2 Institute for Population and Development Studies, School of Public Policy and Administration, Xi’an Jiaotong University, Xi’an 710049, China; shzhli@mail.xjtu.edu.cn; 3 Morrison Institute for Population and Resource Studies, Stanford University, Stanford, CA 94305, USA; mfeldman@stanford.edu

**Keywords:** socioeconomic status, institutional power, market transition, body mass index, obesity

## Abstract

Despite the vast literature on the socioeconomic status (SES) gradient of obesity among adult people, no study has investigated the relationship between institutional power and body mass index. Using national survey data from the “China Labor-force Dynamics Survey 2016” (CLDS 2016), multistage cluster-stratified probability proportional to size (PPS) sampling was employed to select cases from 29 provinces, cities, and autonomous regions in China. This study adopts an institutional approach to explore the influences of SES and institutional power on the state of being overweight or severely overweight (obese) among Chinese adults. It is shown that SES has a non-linear influence on being overweight or obese, higher education has a negative effect on being overweight or obese, income has an inverted U-shaped effect on being overweight or obese, and having a managerial or administrative job has a positive effect on being overweight but less so on obesity. These findings reveal that disparities in health outcome and risks are due to inequality in SES. The work unit is a stronger predictor of adults being overweight or obese than occupation. Working in the public sector has a positive effect on being overweight relative to working in the private sector, and only state institutions and government departments have a positive association with obesity. Our results indicate that institutional structure still has effects on individuals’ life chances in the era of China’s market transition.

## 1. Introduction

The increasing prevalence of being overweight or obese has become a major global public health problem [1,2], due to health consequences, such as hypertension and type 2 diabetes [3,4], and has attracted the attention of many scholars [5,6,7,8]. Studies on being overweight or obese in developed countries have consistently found a negative relationship between socioeconomic status (SES) and being overweight or obese [7,8,9,10,11]. In contrast, research in developing countries has produced inconsistent findings on the relationship between SES and being overweight or obese. Data from poorer countries show a positive relationship between obesity and SES [8,12,13,14,15,16,17]. However, in some middle-income countries, there is a negative association between obesity and SES [5,6]. These findings from developing countries reveal that the relationship between SES and obesity is moderated by the level of gross domestic product [15], and this pattern may exists even inside one country [16,17].

China has experienced a rapid increase in the prevalence of being overweight or obese, and increasing inequality in SES due to more rapid economic growth and urbanization than experienced in most Western countries. Many scholars examined the association between SES and being overweight or obese as a potentially important cause of disease, but the findings have been inconsistent [18,19,20,21,22,23]. Some studies suggested that individuals with higher household income or lower education were more likely to be obese [18,19,20,21]. Other studies showed that not only is there an increasing trend of obesity in all SES groups, which is more dramatic in low SES residents, but high SES residents had slightly decreasing trends in BMI and obesity over the same period [22,23]. One study also found that the relationship between parent’s SES and children’s BMI has changed from positive to negative, with the development of social economy [24]. These inconsistent findings on the disparity in obesity in China might be due to variation in the stages of industrialization between different regions, and in different SES groups. However, existing literature has paid little attention to this issue.

Sociological institutionalism suggests that variation in BMI in China should be investigated from an institutional perspective. The core idea of sociological institutionalism is that institutions shape individuals’ social identification and guide their social behavior [25]. Although the scale of market sectors in China is growing with market reforms, the state (public) sectors still maintain an advantage in the process of resource distribution. In contemporary China, the institutional power possessed by work units contributes to more social inequality than the positional power attached to individuals [24]. Compared to the private sector, state sectors still continue to play an important role in determining their employee’s economic wellbeing due to their redistributive power [26,27]. One study showed that parental state sector employment has a negative influence on a child’s BMI [24]. To our knowledge no study of the effects of work units with institutional power on being overweight or obese has been carried out on the adult Chinese population.

This study aims to explore the effects of both SES and institutional power on being overweight or obese using data from the “China Labor-force Dynamics Survey 2016” (CLDS 2016). To explore the effect of SES on obesity, our analysis includes education, income, and occupation. To explore the effect of institutional power on obesity, our analysis includes the type of work unit, namely the public sector, which includes the state sector (such as government agencies, state institutions, state-owned firms) and collective firms, private sector enterprises, and non-work units (such as self-employment).

### 1.1. Socioeconomic Status and BMI in China

The literature offers several theoretical mechanisms for the link between SES inequality and body mass index (BMI). These mechanisms have been called “material/structuralist”, “cultural/behavioral”, and “psychosocial” [9,28]. The material or structuralist explanation centers on the way that SES may influence health status through its effect on access to health-promoting resources [29]. Income is the basic quantity associated with BMI. Numerous studies have shown a consistently inverse relationship between income and BMI in developed countries [9,30]. However, there is a different situation in low and middle-income countries. The relationship between SES and obesity is positive in developing countries, especially in low-income countries. People in lower SES groups lack food [31], and may not own a vehicle [32], both of which would protect against obesity. However, individuals of higher SES can obtain adequate energy-dense food, which leads to an increase in energy intake and to weight gain due to the cultural value that fat body shape is regarded as a sign of health and wealth [31,32,33]. The relationship between SES and obesity is more complex in middle-income countries, where increasing evidence supports a negative association between SES and obesity [5], but other studies from a middle-income country also found that there is a positive association between SES and obesity in the low-income group [16,34]. Thus, two opposite relationship patterns between SES and obesity can exist within the same country in some developing countries. 

As the largest developing country, China has undergone more rapid economic growth and urbanization than experienced in most Western countries. This has not only led to great changes in nutrition and lifestyle, but has also increased inequality in SES. The middle and high SES groups have more resources to spend on healthy food, while the low SES groups have difficulty in acquiring healthy food. Increasing income would be associated with increasing obesity among the low-income population. However, increasing income would be associated with decreasing obesity among the middle- and high-income populations. Therefore, we suggest our first hypothesis.

**Hypothesis** **1.** *There is an inverted U-shaped relationship between income and being overweight or obese.*

The cultural or behavioral explanation posits that SES may exert its effects on health through lifestyles and behaviors, such as patterns of diet, sleep, exercise, smoking, drinking, and drug use [28,29]. Education is an important contributor to individual lifestyle and health behavior. Although numerous studies have documented the inverse relationship between education and obesity [9,35], the level of education may be more important than the number of years of education. Because schools in China focus on examination-oriented education, and seldom offer other knowledge through elementary and high school education, people without higher education might lack enough knowledge about health to observe a healthy lifestyle. Thus, we suggest the second hypothesis.

**Hypothesis** **2.** *There is an inverse relationship between level of education and being overweight or obese in the population with higher education.*

The psychosocial explanation emphasizes that the role of SES in the distribution of health status is due to differences in exposure to psychosocial stress, which might con-tribute to an unhealthy lifestyle [28]. Some literature shows that there may be a positive relationship between job strain and BMI [36], while other studies find that the more job stress, the lower the BMI [37]. Yet, other studies find no relation between job stress and BMI [38]. Two explanations have been suggested for these inconsistent findings. The first is that psychosocial stress may reduce appetite, which might lead to weight loss [39,40]. The second is that work stress might lead to an unhealthy lifestyle [41], such as physical inactivity [42] and poor diet [43], which might result in weight gain. However, the second explanation is derived from samples in developed countries. A study from China found that there is a positive association between subjective well-being and BMI [44]. Lower occupational status is linked to some job stress, such as low job control [45] or job insecurity [46], which are associated with low level of happiness and high rate of weight loss. Thus, we suggest a third hypothesis:

**Hypothesis** **3.** *There is a positive relationship between occupational status and being overweight or obese.*

### 1.2. Institutional Power and BMI in China

Although SES inequality in BMI has been found in numerous empirical studies, sociological institutionalism suggests that variation in BMI in China should be investigated in the context of market transformation. First, China’s market reform has increased the scale of market sectors, but marketization does not remove the advantage of political power [47]. As central institutions of political control, work units are assigned different priorities according to their position in the redistribution economy, which determines individuals’ entitlement to private benefits and public goods [24,27,47]. Second, with marketization, deinstitutionalization in labor standards has taken place in China, and the greater the extent of marketization the more deinstitutionalization takes place. Compared to public sector work units, private sector work units are more likely to adopt deinstitutionalized employment practices to reduce costs and to promote flexibility, which they can do in the absence of independent unions and the states’ failure to enforce labor laws [48]. 

These shifts have two kind of consequences: on the one hand, public sector work units have advantages in income and in other benefits, such as job security, pensions, and medical insurance over private sector work units [49]. On the other hand, workers in the private sector are more vulnerable to trends in deinstitutionalization than those in the state sector, due to the absence of independent unions, the government’s default on enforcing labor law, and the employer’s economic motivation [48]. Both of these would be able to create better subjective well-being [49], which may increase appetite [44], and lead to weight gain in public sector.

Work units also shape people’s lifestyle, which is associated with BMI. “*Guanxi*” (social connection) is an important way to acquire power, status, and resources in China [50], and valuable *guanxi* is also related to work units. The person in the work unit that is closer to the power of redistribution, is likely to have more valuable guanxi. Such tactics as presenting gifts and holding banquets for the other party are used to build and enhance *guanxi* in Chinese society [51]. A person in a state sector work unit has more opportunity to hold a banquet for the other party and participate in a banquet and might, therefore, be at risk for being overweight or obese: such frequent social interactions not only lead to eating more high energy food but also to a reduction in the leisure time available for physical activities. Thus we suggest a fourth hypothesis.

**Hypothesis** **4.** *Compared to the private sector, the state sector has higher rates of being overweight or obese.*

Some studies suggest that although public sector work units continue to take advantage of the power of redistribution and play an important role in shaping employees’ incomes, disparities exist between work units [26]. State-owned and collective firms face more pressure to deinstitutionalize than other state sectors, such as government and public institutions, due to the development of marketization. Compared to government agencies and state institutions, state and collective firms are facing increasing competition from the market, which can marginalize these work units [26]. Thus we suggest a fifth hypothesis:

**Hypothesis** **5.** *There are differences in influence on obesity between sections of the public sector, the influences of government and state institutions on obesity are more significant than other work units in the public sector.*

## 2. Data and Methods

### 2.1. Sample 

All analyses for this study use data from the “China Labor-force Dynamics Survey 2016” (CLDS 2016), the first comprehensive national survey targeting at the labor force in China. The survey used multistage cluster-stratified probability proportional to size (PPS) sampling to select cases from 29 provinces, cities, and autonomous regions in China, which ensures that the sample in CLDS can be regarded as a nationally representative sample. The provinces were stratified by the size of their population and labor force, and all regions were divided into eight strata to constitute a total national sample after weighting [52]. A detailed description of the design and implementation of the survey can be found in Wang’s study [52]. Targeted respondents in the survey are civilians aged from 15 to 64. CLDS2016 interviewed 21,086 respondents. 

To investigate the influence of work units on BMI among Chinese adults, this study selected respondents according to the following criteria. First, suitable respondents should be active in labor market, that is, they were required to have a job (including farming, part-time work, and helping in the family business); the sample size was 14,548. This wave survey interviewed about 6436 respondents who were identified as farmers, but farming implies a high degree of self-sufficiency outside of the market in China. In order to delete respondents who were identified as self-sufficient farmers, we selected respondents who also had wage income in the labor market; this sample size was 8260. In addition, we selected respondents aged from 18 to 64 as the study subjects, this sample size was 7913. Because the village committee is neither in the market sector, nor in the public sector, we deleted respondents who work in village committee, leaving a sample size of 7083. Following the deletion of respondents who were identified as soldiers by profession, a sample size of 7059 was obtained. The final total sample size in the study was 6592, because there were some missing data. 

### 2.2. Measures

#### 2.2.1. Dependent Variables 

WHO defines BMI ≥ 25.0 kg/m^2^ as being overweight and BMI ≥ 30.0 kg/m^2^ as being obese. However, because of the difference in body fat content between East Asian and Europeans, many studies use the Chinese standard BMI classifications for adults [20]. In the present study, being overweight was defined as BMI ≥ 24 kg/m^2^ and obesity was defined as BMI ≥ 28 kg/m^2^ for adults [20]. 

#### 2.2.2. Independent Variables 

Socioeconomic status was measured by education [9], income [9], and occupation [53].

Educational attainment was measured as a categorical variable (middle school or below, high school, vocational-technical school, junior college, bachelors degree or higher). 

Annual disposable personal income was measured as a continuous variable equal to the difference between total annual personal income, which includes wage income, agricultural income, operating income, bonuses and other subsidies, and charges that include individual income tax, social insurance charges, and the housing accumulation fund. 

Occupational class was defined using five categories: professional manager, professional and technical personnel, clerical staff, business services personnel, and manual worker and other. 

Work units were measured using six categories, consistent with previous research on the market transition [26,54]: government agencies (*jiguan*), state institutions (*shiye danwei*, e.g., schools, research centers, medical facilities, etc.), state-owned firms (*guoying qiye*), collective firms (*jiti qiye*), non-work units (*wu danwei*, e.g., peasant, stall-keeper, self-employed driver, etc.), and private sector firms (e.g, domestic private companies, foreign-invested firms, international joint ventures, state-private or collective-private joint ventures, etc.). Previous studies have often employed a dichotomous classifications of work units into public versus private sectors [26], where the private sector includes several organizational forms [24]. For example, self-employment could be classified into non-work units, and domestic private firms could be classified as private sector firms. Thus, the six categories can be grouped into three types: public sectors (government agencies, state institutions, state-owned firms, and collective firms), private sector firms and non-work units.

#### 2.2.3. Control Variables

The following socio demographic control variables were used: age, gender, marital status, work pressure, lifestyle [9], neighborhood integration [10], social welfare [45], Hukou [44], and location area [24],

Age was measured in years.

Gender was recorded as a dummy variable (male = 0, female = 1).

Hukou was used to control disparity between urban and rural residence, and was recorded as a dummy variable (urban = 1, rural = 0).

Location areas were used to control regional disparity. It was measured as a dummy variable (coastal region = 0, middle region = 1, western region = 2).

Two dummy variables were used to control for the disparity in social welfare: housing accumulation fund (have = 1, have not = 0); medical insurance (have = 0, have not = 1).

Marital status is a dummy variable (First marriage, remarriage, or cohabitation = 0, Single = 1, Divorced or widowed = 2). Age is a continuous variable (from 18 to 64).

Four dummy variables were used to measure lifestyles. Regular exercise is a dummy variable (yes = 0, no = 1). Number of cigarettes smoked per day is a dummy variable (no smoking = 0, less than 10 = 1, 10 to 19 = 2, 20 and more = 3). Rate of drinking is a dummy variable (no drink or once every few weeks = 0, 1–2 times every week = 1, 3–4 times every week = 2, drink every day = 3). Rate of social engagement is a continuous variable (3–15) and included three questions. Respondents were asked, “How often did you invite other people to have lunch or dinner in a restaurant in the past three months”, “How often were you invited to have lunch or dinner in restaurant in the past three months”, “How often did you have lunch or dinner in restaurant with friends in the past three months”. Responses were coded from 1 (never) to 5 (often), and scores are the sum of the responses on the three items ranging from 3 to 15. The higher the respondent’s score the higher the frequency of social engagement.

Two dummy variables were used for work pressure. Workload or work intensity was used to measure job autonomy, which is a dummy variable (independent = 0, partly dependent on another person’s decision = 1, entirely dependent on another person’s decision = 2). Job status is a dummy variable (full-time job = 0, part-time job = 1). Working hours per week is a dummy variable (40 h or less = 0, 41 to 50 = 1, 51 to 60 = 2, 61 and more = 3).

Finally, familiarity with neighbors was measured to indicate neighborhood integration and was a dummy variable (unfamiliar = 0, familiar = 1).

### 2.3. Data Analysis

Our study was conducted in three steps. First, descriptive statistics were used to analyze the relationship between SES, work unit, and being overweight or obese. Second, to explore the difference in welfare between the private and public sector, we used contingency tables to study the relationship between work-unit welfare and work units. Third, bivariate and multinomial logistic regression were often used to estimate the predictors of being overweight or obese [55,56,57]. To explore associations between SES, work units, and adults being overweight or obese, we used multinomial logistic regression models.

We employed three models for this analysis: first, we adjusted for control variables, and examined the association between SES and adult overweight/obesity; second, we adjusted for income, education, and control variables, and measured the association between work units and adult overweight/obesity; third, we adjusted for control variables, and examined the association between socioeconomic status, work units, and being overweight or obese.

## 3. Results

### 3.1. Descriptive Statistics for the Relationship between SES, Work Units, and Being Overweight or Obese

Table 1 reports the descriptive statistics for variables in this study and shows that 26.56% of 557 adults were overweight, whereas about 8.10% were obese. Thus being overweight and obesity are prevalent health problems among the adult Chinese population.

Table 2 shows the BMI distribution according to occupation, work units, and education. There is a significant difference in BMI according to occupation, education, and work unit (*p* < 0.001). The descriptive statistics for occupation by BMI shows that the manager group has the highest rate of being overweight and lowest rate of normal weight or being underweight; clerical staff and business services personnel groups have the two highest rates of obesity; the technology professional group has the lowest rate of being overweight and obese and the highest rate of being normal weight and underweight. For education by BMI, the junior college and the bachelors degree or higher groups have the two highest rates of being normal weight or being underweight, and the two lowest rates of being overweight. The middle school or below and the high school groups have the two lowest rates of normal or below normal weight and the two highest rates of being overweight; the middle school or below and the junior college groups have the two highest rates of obesity. Among work units, the private sector group has the highest rates of normal or below normal weight, a low rate of being overweight, and the lowest rate of obesity. However, compared to the private sector group and the no-work-unit group, collective firms, state-owned firms, state institutions, and government agencies groups have the lowest rates of normal or below normal weight, and the four highest rates of obesity. The government agencies group has the highest rate of obesity. Collective firms, state-owned firms, and state institutions groups have the three highest rates of being overweight.

Table 3 shows the relationship between occupations and education and welfare. Technology professionals, clerical staff, and managers have a higher fraction with higher education than business services personnel and manual workers. There is a positive relationship between occupation and income; managers have the highest income, and manual workers have the lowest. There is also a positive association between occupation and social engagement; managers have the highest rate of social engagement, and manual workers the lowest. In addition, compared to business services personnel and manual workers, clerical staff, technology professionals, and managers have distinctive advantages in their housing accumulation funds, medical insurance, and low rate of prolonged work hours. Thus, high grade occupations are simultaneously facing positive and negative factors that contribute to being overweight or obese.

### 3.2. The Difference in Welfare between Public and Private Sector

We used contingency tables to quantify the relationship between work-unit welfare and work units. Descriptive statistics for the work unit related to welfare are presented in Table 4. There is a significant difference in welfare between the public and private sectors (*p* < 0.001). Compared to employees in the private sector, employees in the public sector not only had higher average annual income, but also a higher rate of medical insurance and a greater housing accumulation fund. Although there is a serious problem of overtime work in both the public and private sectors, it is more serious in the private sector than in the public sector. In addition, employees in the public sector have higher rate of social engagement than those in the private sector.

Table 4 also shows that there is a significant difference in BMI between the private and public sector (*p* < 0.001). The fraction who are normal and below normal in weight is higher in the private sector than in the public sector. More people are overweight and obese in the public sector

Table 5 shows the association between welfare and work unit among public sectors. One-way ANOVA shows that compared to employees in collective firms, employees in government agencies and state institutions have greater income (*p* < 0.05) and more frequent social engagement (*p* < 0.1/*p* < 0.01). Additionally, compared to state-owned firm employees, government agency employees have greater income and more frequent social engagement. State institution employees have more frequent social engagement but slightly less income.

The contingency tables show that compared to collective firm employment, government agencies and state institutions not only have advantages in medical insurance, and housing accumulation fund distribution (*p* < 0.001), but also have lower rates of extended work hours (*p* < 0.001). Compared to state firm employment, government agency and state institution employment also have slight advantages in medical insurance and housing accumulation fund, but only government agencies have a significant advantage in housing accumulation fund (*p* < 0.05). Compared to state-owned firm employment, government agencies and state institutions also have advantages in prolonged work hours (*p* < 0.05/*p* < 0.1), government agencies and state institutions have lower rates of extended work hours. These results show that there are some differences in welfare distribution, with government agencies and state institutions offering more welfare benefits than other work units in the public sector.

### 3.3. The Influence of SES and Work Units on Being Overweight or Obese

To explore the relationship between SES, work units, and being overweight or obese, three models were employed. Table 6 presents logistic regression analyses of being overweight and obese. Model 1 reports the associations between SES and being overweight or obese. Model 2 shows the effects of work units, income, and education on being overweight or obese (This model omits the variable of occupation and adjusts for all control variables). Model 3 shows the relationship between SES, work units, and being overweight or obese (Model 3 adds SES and work unit simultaneously and adjusts for all control variables).

There is an inverted U-shaped relationship between income and being overweight or obese. Model 1 shows a significant positive relationship between income and being overweight and obese (*p* < 0.05/*p* < 0.1), and there is a significant negative relationship between the log of income squared and being overweight or obese (*p* < 0.05). Model 2, which adjusts for work unit and other control variables, shows a similar relationship between income and being overweight or obese. Income has a slightly significant positive effects on being overweight or obese (*p* < 0.1), but log of income squared has a significant negative effect on these (*p* < 0.1). Model 3 adds SES, work unit, and control variables, and shows that income has a weak positive effect, but log of income squared has a weakly significant negative effect on these (*p* < 0.1). Thus, there is an inverted U-shaped relation between income and being overweight or obese, which supports Hypothesis 1.

There is an inverse relationship between higher education and being overweight or obese. Model 1 indicates that, compared to middle school and below, junior college, bachelors degree, and higher have a lower rate of being overweight (*p* < 0.1, *p* < 0.05); high school, vocational-technical school, junior college, bachelors degree, and higher have lower rates of obesity. With Model 2, compared to middle school and below, junior college, bachelors degree, and higher, have a lower rate of being overweight (*p* < 0.1, *p* < 0.01). High school, junior college, bachelors degree, and higher also have lower rates of obesity (*p* < 0.1, *p* < 0.1, *p* < 0.05). Although vocational-technical school has no significant influence on obesity (*p* > 0.1), it does appear to slightly reduce the rate of obesity. In Model 3, we add SES, work unit, and the control variables, and the results show that junior college, bachelors degree, and higher have slightly significant negative effects on being overweight (*p* < 0.1, *p* < 0.05). High school, junior college, bachelors degree, and higher have weakly significant negative effects on obesity (*p* < 0.1, *p* < 0.1, *p* < 0.1). The effect of vocational-technical school is not significant (*p* > 0.1). These findings reveal that, although intermediate educational level has no significant influence on being overweight, compared to middle school and below, more education has a significant negative effect on being overweight. The results also show that compared to middle school and below, high school and higher education have significant negative effects on obesity. These results support Hypothesis 2.

Manager or administrative jobs and business services jobs have significant positive associations with being overweight, but only business services jobs have a significant positive association with obesity. Model 1 shows that, compared to manual jobs, business services jobs have significant positive effects on being overweight and obese (*p* < 0.05, *p* < 0.01), and manager or administrative jobs have significant positive effects on being overweight (*p* < 0.05). Model 3 adds SES, work unit, and other control variables and shows that business services jobs have significant positive effects on being overweight and obese (*p* < 0.1, *p* < 0.05), and manager or administrative jobs have significant positive direct effects on obesity (*p* < 0.05). Clerical staff jobs and technology professional jobs have no significant effects on being overweight or obese. These results do not support Hypothesis 3.

Compared to SES, work unit is a stronger predictor of adult being overweight or obese. Model 2 shows that compared to the private sector, collective firms, state-owned firms, and state institutions have positive effects on being overweight (*p* < 0.05). State institutions and government departments have positive effects on obesity (*p* < 0.05, *p* < 0.01). Model 3 adds SES to Model 2 and shows that, compared to the private sector, collective firms, state-owned firms, and state institutions still have significant positive effects on being overweight, and state institutions and government department have significant positive effects on obesity (*p* < 0.01). Thus, compared to the private sector, public sector work units have higher rates of being overweight and obesity, which supports Hypothesis 4. However, there is a difference in the rate of obese among state sectors. State institutions and government agencies have significant positive effects on obesity (*p* < 0.01), while collective firms and state-owned firms have no significant effects on obesity (*p* < 0.1). These results support Hypothesis 5.

## 4. Discussion

Although research is beginning to address the determinants of being overweight or obese among adults in China, most studies have used regional survey data and have focused on the relationship between SES and being overweight or obese. There has been little exploration of the association between work unit and being overweight or obese. This study addresses the impact of work unit and SES on these from an institutional perspective, exploring the relationship between social stratification and health in China’s post-market transition era. Our results suggest that SES has a non-linear influence on being overweight and obese, and work unit has strong positive effects on being overweight and obese.

The first hypothesis (H1) that there is an inverted U-shaped relationship between income and being overweight or obese was supported, which is consistent with some studies in middle income countries [5,16]. The result not only tallies with the conjecture made for middle-income countries by Fernald [16], but is also consistent with the findings of Esposito et al. [34] and Quezada and Lozada-Tequeanes [58]. Higher income is associated with a higher rate of being overweight or obese among low-income groups; however, higher income is associated with a lower rate of being overweight or obese in middle and high income groups. These findings are also consistent with a previous study in the Chinese context that lower middle class are likely to have a higher BMI than the poor and the new middle class (upper middle class) has a lower probability of being obese [59]. This suggests that these opposite relationships between income and being overweight or obese in China reflect the ongoing nutritional transition. According to material or structuralist explanations, those living in extreme poverty may not have enough resources to gain weight [60], and low-income people with more resources are more likely to consume a high energy diet than vegetables and fruits to save money [61], which contributes to their being overweight or obese. However, people with high income can afford more healthy food [62], which is also related to maintaining a desirable body weight. The findings further establish the importance of structural advantages or disadvantages related to acquisition of material resources in determining health inequalities among the Chinese adult population.

Our second hypothesis (H2), that there is an inverse relationship between education and being overweight or obese among the population with higher education is supported. We found that compared to non-diploma levels of education, higher education has a weak negative effect. This is consistent with prior studies in developed and middle income countries where education has been shown to have a protective function against obesity [63,64]. Higher education may help people to understand the negative consequences of obesity, and help to lead a healthy lifestyle [65]. Education in a vocational-technical school has no significant effect on being overweight or obese, which indicates that there is a non-linear relationship between level of education and being overweight or obese. These finding highlight the importance of higher education on being overweight or obese among adults in China.

The third hypothesis (H3) that there is a positive relationship between occupational status and being overweight or obese is not supported. We found that there is a non-linear relationship between occupation and being overweight or obese in Chinese adults. First, compared to manual work, business services work has a significant positive effect on being overweight or obese. Second, compared to manual work, being a manager has a significant association with being overweight, but not with being obese. Although these are not consistent with reports that in high and middle income countries higher SES is associated with lower BMI [66], it is in line with findings in several studies in USA and Mexico that elite strata are at risk of weight gain, but the prevalence of obesity among this group is relatively low [53]. Third, compared to manual workers, being clerical staff or technology professionals has no significant effect on being overweight or obese. Possible reasons for this could be that, in our case, business services workers have higher income than manual workers but are less likely to have higher education, which means that increase in income is not accompanied by an increase in knowledge [53], but might allow them to consume more high-calorie foods, which then contributes to weight gain [16]. We also found that managers have the highest level of income and highest frequency of social engagements. The higher level of income may allow the managers to purchase healthier food, but their high frequency of social engagement may make them take in more calories. Both might lead managers to be overweight but not obese. In addition, the higher level of education among clerical staff or technology professionals may help to explain why the association between clerical staff or technology professionals and being overweight or obese is insignificant. These findings show that career-orientated lifestyles can be related to indirect factors of weight gain such as material well-being, resulting in an unhealthy way of life [67].

The fourth hypothesis (H4) that compared to those in the private sector, workers in the public sector should have a high rate of being overweight or obese is supported. We find that working in the public sector has a positive effect on being overweight relative to the private sector. Working in collective enterprises and state-owned enterprises and state institutions has a positive relationship with being overweight, while state institutions and government departments are positively associated with obesity. These results are consistent with the finding of Fu and George that the public sector still has distinct advantages [24]. Here, are some possible reasons for the public sector’s positive effect. The first is the influence of deinstitutionalization due to marketization [48]. Since the market transition, deinstitutionalization has occurred in both the private and state sectors in the course of profit maximization. Because of fierce market competition, the goal of profit maximization, and the absence of independent unions [48], the private sector is more likely to deinstitutionalize work patterns in order to reduce labor costs and maximize productivity. This not only causes employees to encounter more fierce competition from the market, but also to suffer more serious work stress, which might decrease appetite and lower calorie intake. Employees in the market sector confront more fierce market competition, which may increase the demand for overtime, increase physical activity, and thus burn more calories. Under these conditions employees in the market sector would be protected against a high rate of being overweight or obese. However, compared to the private sector, the influence of deinstitutionalization in the public sector is weak. Due to the leadership of the Chinese Communist Party and the ideology of socialism, public sector employees not only have a more egalitarian welfare distribution than those in the private sector [54,68], but also have a higher level of job security and stability, which might result in public sector employees having better subjective well-being [49], and maintaining a good appetite [44,69]. The result would be higher rates of being overweight or obese than among employees in the private sector.

In addition, the social context of the interactive relationship between the Chinese culture of human relationship and the power of redistribution may partially explain the different rates of being overweight or obese between the public and private sectors. On the one hand, the public sector still controls core resources and has the advantage of being able to determine resource redistribution. This gives employees in the public sector positional advantages in the resource redistribution system and also endows people who have redistribution power with the role of broker. On the other hand, building human relationships (*guanxi* in Chinese) and using these relationships are the main informal ways that social resources are accessed in China, especially in the public sector [55], where the degree of marketization is low [25]. Giving gifts and holding banquets are the main strategies to build and enhance *guanxi* in Chinese society [52]. Therefore, compared to employees in the private sector, those in the public sector have more opportunities to hold banquets. Both types of social activity make employees in the public sector more likely to increase their calorie intake, which can result in higher rates of being overweight and obese than employees in the private sector. The results in Table 4 support this explanation. Compared to the private sector, the public sector is not only better at conducting the social welfare that is stipulated by the labor laws, and hence is better able to protect public sector employees’ social welfare, but also has a higher rate of social activity than employees in the private sector. Meanwhile, the negative effects of work units on BMI also show that they not only still play an important role in the distribution of life chances and social welfare, but also shape employees’ lifestyles and social activity, according to their position in the redistributive hierarchy.

The fifth hypothesis (H5), that there are work unit differences in influence on obesity within the public sector is supported: the influences of government and state institutions on obesity are more significant than other work units in the public sector. We find that compared to the private sector, collectively-owned and state-owned enterprises do not, but state institutions and government agencies do have significant effects on obesity. This difference might also be associated with the process of deinstitutionalization in the public sector. Under market conditions, collectively-owned and state-owned enterprises are confronted with fierce competition from the private sector [27], which also motivates the former to adopt deinstitutionalized work patterns to reduce costs [49]. It also may lead people who work in the collectively-owned and state-owned enterprises to suffer from more work stress, which might lower their appetite and prevalence of obesity. Table 5 shows that compared to collectively-owned and state-owned enterprises, workers in state institutions and government agencies not only receive more social welfare, but also have a higher frequency of social engagement, which might result in a high rate of obesity. Thus, although both state-owned and collective enterprises still have some advantages over the private sector, these advantages have been undermined by the growth of the private sector. However, state institutions and government agencies are close to the center of redistribution power [27], which enables these work units to offer advantages over the private sector and both state-owned and collective enterprises [27]. Thus, future studies on health inequality need to take into account the disparity among the various public sectors.

## 5. Conclusions

We have demonstrated that socioeconomic status is related to health outcomes in the present era of market transition. We found that there is a non-linear relationship between SES and being overweight or obese. Income has an inverted U-shaped effect on being overweight or obese, while higher education has a weak negative effect on being overweight and obese. These results indicate that disparities in health outcome and risks are due to inequality in SES. In addition, the type of work unit has a robust association with being overweight or obese, and the relationship between the type of work unit and being overweight or obese differs with the position in the hierarchy of work units. Government and state institutions are located at the center of the redistribution system and offer more social welfare advantages than other work units, which contributes to the high rate of obesity among the employees in these sectors. Our results suggest that state power still plays an important role in the social stratification process, which not only affects socioeconomic inequality [28], but also shapes inequality in health status.

## 6. Limitations

Our study is subject to several limitations. First, we use self-reported weight and height to compute BMI due to the CLDS survey’s limitations. Second, we are unable to explore occupational differences in being overweight or obese between the state sector and private sectors because of the sample size, which does not allow us to analyze the effects of occupation on being overweight or obese separately for state and private sectors

Despite its limitations, this study enriches the literature in two ways. First, most studies that address these topics focus on the relationship between SES and obesity; and overlook the influence of institutional power on adult obesity. This study explores the influence of the work-unit system on disparity in obesity among the adult Chinese population and reveals that institutional power may have a negative effect on bodyweight management. This, may have implications for the role of social stratification in health disparities in some non-Western societies. Second, this study shows that there is a non-linear relationship between SES and being overweight or obese in China. This finding is not only inconsistent with findings in previous studies in developed countries that there is negative association between SES and being overweight or obese [9,12], but is also different from results that were found for low-income countries [8,14]. Our findings show that the nutritional transition is more complex in China than in developed countries, and more specific and targeted public health programs and policies should be designed to increase healthy behaviors in an all work-unit.

## Figures and Tables

**Table 1:** Descriptive statistics for variables used in analysis of BMI (N = 6592).

Type	Mean/SE	Type	Mean/SE
Body Mass		Self -reported health	
Normal/underweight (Less than 24)	0.65/0.01	Healthy	0.67/0.01
Overweight (24 to less than 28)	0.27/0.01	Fair	0.26/0.01
Obesity (28 and more)	0.08/0.00	Unhealthy	0.07/0.00
Socioeconomic Status			
Education		Work hours	
Middle school or below	0.56/0.01	40 h or less	0.42/0.01
High school	0.14/0.00	41 to 50	0.18/0.01
Vocational-technical school	0.07/0.00	51 to 60	0.19/0.01
Junior college	0.11/0.00	61 and more	0.21/0.01
Bachelor degree/higher degree	0.12/0.00	Workload/work intensity	
Income	37,847/45,163	Independent	0.35/0.01
Lognormal of income	10.11/1.02	Dependent on other’s decision partly	0.31/0.01
Occupation		Dependent on other’s decision entirely	0.34/0.01
Manager	0.01/0.00	Job status	
Technology professional	0.11/0.00	Full-time job	0.88/0.00
Clerical staff	0.05/0.00	Part-time job	0.12/0.00
Business services personnel	0.35/0.01	Housing accumulation fund	
Manual worker and other	0.47/0.01	Have not	0.78/0.01
Institutional Power		Have	0.22/0.01
Work units			
Private sectors	0.48/0.06	Medical insurance	
No work unit	0.28/0.01	Have	0.47/0.01
Collective enterprises	0.02/0.00	Have not	0.53/0.01
State enterprises	0.07/0.00	The number of cigarettes smoked	
State institutions	0.11/0.00	No smoking	0.67/0.01
Government agencies	0.04/0.00	Less than 10	0.06/0.00
Gender		10 to 19	0.09/0.00
Male	0.56/0.01	20 and more	0.18/0.01
Female	0.44/0.01	Frequency of drinking alcohol	
Age	41.60/11.38	No drink/once every few weeks	0.75/0.01
Hukou		1–2 times every week	0.13/0.00
Rural	0.52/0.01	3–4 times every week	0.05/0.00
Urban	0.48/0.01	Drink every day	0.07/0.00
Marriage status		Frequency of social engagement	6.64 /3.07
First marriage/remarriage/cohabitation	0.82/0.01	Regular exercise	
Single	0.13/0.00	Yes	0.36/0.01
Divorce, widowed,	0.05/0.00	No	0.64./0.01
Area		Familiarity with neighbors	
Coastal region	0.23/0.01	Unfamiliar	0.15/0.00
Middle region	0.19/0.01	Fair	0.28/0.01
Western region	0.58/0.01	Familiar	0.57/0.01

**Table 2:** Difference in BMI by main independent variable (100%).

	BMI (Percentages)			*p*-Value
	Normal/Underweight	Overweight	Obesity	
Occupation				
Manager	53.57	38.10	8.33	***
Technology professional	72.12	21.22	6.66	
Clerical staff	64.71	25.49	9.80	
Business services personnel	63.93	26.82	9.24	
Manual worker and other	65.20	27.42	7.39	
Work units				
Private sectors	68.94	24.21	6.85	***
No work unit	63.98	27.53	8.49	
Collective enterprises	53.21	36.70	10.09	
State enterprises	57.98	33.61	8.40	
State institutions	60.84	29.23	9.93	
Government agencies	61.28	23.83	14.89	
Education				
Middle school or below	64.29	27.48	8.23	***
High school	61.51	31.03	7.46	
Vocational-technical school	65.82	26.10	8.08	
Junior college	69.40	22.28	8.32	
Bachelor degree/higher degree	70.68	21.25	8.07	
Income (yuan) ^a^	37,874.43	37,807.04	37,761.87	

Note: significance level: *** *p* < 0.001; ^a^: the figure denotes mean.

**Table 3:** Difference in education and welfare by occupation (100%).

		Occupation (Percentages)					*p*-Value
		Manager	Technology Professional	Clerical Staff	Business Services Personnel	Manual Worker and Other	
Education	Middle school or below	22.62	6.51	14.53	48.63	79.02	
	High school	16.67	9.00	19.27	18.47	11.24	***
	Vocational-technical school	7.14	10.94	6.15	8.24	4.37	
	Junior college	23.81	27.01	24.30	13.91	3.93	
	Bachelordegree/higher degree	29.76	46.54	35.75	10.57	1.44	
Income (yuan) ^a^		91,709.50	55,357.38	48,345.44	42,770.31	27,488.83	***
Social engagement ^b^		8.27	7.99	7.90	7.27	5.68	***
Housing accumulation fund	Have	47.62	52.70	64.99	21.79	9.02	***
	Have not	52.38	47.30	35.01	78.21	90.98	
Medical insurance	Have	73.81	81.55	90.76	55.82	26.87	***
	Have not	26.19	18.45	9.24	44.18	73.13	
Work hours	40 h or less	61.90	66.57	68.91	39.37	35.12	
	41 to 50	10.71	20.11	13.17	21.61	15.67	***
	51 to 6	14.29	7.63	9.24	17.75	23.86	
	61 and more	13.10	5.69	8.68	21.27	25.34	

Note: significance level: *** *p* < 0.001; ^a^ and ^b^: the figure denotes mean.

**Table 4:** Differences between public and private sectors in welfare, social engagement, work hours, and BMI (100%).

	Type of Work Unit (Percentages)		Difference	Pr		Type of Work Unit (Percentages)		Difference	*p*-Value
	Public Sector	Private Sector				Public Sector	Private Sector		
Income (yuan) ^a^	48,091.52	41,754.38	6337.14	***	Work hours				
Medical insurance					40 h or less	65.93	30.84	35.09	***
Have	86.25	48.33	37.92	***	41 to 50	15.83	22.61	−6.78	
Have not	13.75	51.67	−37.92		51 to 60	9.90	22.21	−12.31	
Housing accumulation fund					61 and more	8.34	24.34	−16.00	
Have	62.41	14.61	47.8	***	BMI				
Have not	37.59	85.39	−47.80		Normal and underweight	59.48	68.94	−9.46	
Social engagement					Overweight	30.29	24.21	6.08	***
The frequency of social engagement ^b^	7.44	6.99	0.45	***	Obesity	10.23	6.85	3.38	

Note: significance level: *** *p* < 0.001; ^a^ and ^b^: the figure denote mean.

**Table 5:** The difference in welfare among public sectors (100%).

		Public Sectors (Percentages)				*p*-Value
		Collective Enterprises	State Enterprises	State Institutions	Government Agencies	
Incomes (yuan) ^a^	Incomes	33,948.62	49,495.99	48,016.22	52,035.74	**
Medical insurance	Have	67.89	86.34	88.34	88.09	***
	Have not	32.11	13.66	11.61	11.91	
Housing accumulation fund	Have	20.18	62.18	66.57	69.79	***
	Have not	79.82	37.82	33.43	30.21	
Social engagement ^b^	Frequency of social engagement	6.67	7.37	7.46	7.91	**
Work hours	40 h or less	44.95	62.39	69.23	72.77	***
	41 to 50	27.52	17.65	14.83	9.79	
	51 to 60	10.09	10.71	9.65	8.94	
	61 and more	17.43	9.24	6.29	8.51	

Note: significance level: *** *p* < 0.001, ** *p* < 0.01; ^a^ and ^b^: the figure denote mean.

**Table 6:** Logistic regression analyses for being overweight and obese (*n* = 6592).

	Model 1		Model 2		Model 3	
	Overweight	Obese	Overweight	Obese	Overweight	Obese
Education (middle school and below = 0)						
High school	0.07 ^a^	−0.27+	0.06	−0.27+	0.06	−0.27+
	(0.09) ^b^	(0.15)	(0.09)	(0.15)	(0.09)	(0.15)
Vocational-technical school	0.03	−0.11	0.001	−0.13	0.01	−0.11
	(0.13)	(0.21)	(0.13)	(0.21)	(0.13)	(0.21)
Junior college	−0.21+	−0.25	−0.23+	−0.34+	−0.22+	−0.31+
	(0.12)	(0.19)	(0.12)	(0.18)	(0.12)	(0.19)
Bachelors degree and higher degree	−0.30 *	−0.33	−0.35 **	−0.50 *	−0.32 *	−0.42+
	(0.14)	(0.21)	(0.13)	(0.21)	(0.08)	(0.13)
Log of income	0.70 *	1.10+	0.60+	1.04+	0.64+	1.042+
	(0.34)	(0.57)	(0.34)	(0.57)	(0.34)	(0.57)
log of income squared	−0.04 *	−0.06 *	−0.03+	−0.06+	−0.03+	−0.056+
	(0.02)	(0.03)	(0.02)	(0.03)	(0.02)	(0.03)
Occupation (manual worker and others = 0)						
Business services personnel	0.15 *	0.35 **			0.13+	0.35 **
	(0.07)	(0.12)			(0.07)	(0.12)
Clerical staff	−0.03	0.23			0.01	−0.18
	(0.15)	(0.22)			(0.17)	(0.27)
Technology professional	−0.02	0.02			−0.06	−0.09
	(0.13)	(0.20)			(0.13)	(0.21)
Manager	0.48+	0.21			0.50 *	0.17
	(0.25)	(0.43)			(0.25)	(0.43)
Work units (private sector = 0)						
No work unit			−0.04	0.18	−0.0008	0.27+
			(0.09)	(0.14)	(0.09)	(0.15)
Collective firms			0.57 **	0.56	0.57 **	0.54
			(0.22)	(0.34)	(0.22)	(0.35)
State-owned firms			0.25 *	0.11	0.25 *	0.09
			(0.12)	(0.20)	(0.12)	(0.20)
State institution			0.22 *	0.41 *	0.26 *	0.48 **
			(0.11)	(0.18)	(0.12)	(0.18)
Government agencies			−0.09	0.69**	−0.07	0.88 **
			(0.18)	(0.23)	(0.20)	(0.27)
Gender (male = 0)	−0.56 ***	−0.63 ***	−0.55 ***	−0.60 ***	−0.54 ***	−0.60 ***
	(0.08)	(0.12)	(0.08)	(0.12)	(0.08)	(0.12)
Age	0.02 ***	0.01	0.01 ***	0.004	0.01 ***	0.005
	(0.003)	(0.005)	(0.003)	(0.006)	(0.003)	(0.006)
Hukou (rural = 0)	0.12	0.04	0.14+	0.13	0.11	0.05
	(0.08)	(0.12)	(0.08)	(0.12)	(0.14)	(0.22)
Marriage status (First marriage, remarriage, cohabitation = 0)						
Single	−0.77 ***	−0.49 **	−0.77 ***	−0.48 **	−0.76 ***	−0.47 **
	(0.12)	(0.18)	(0.12)	(0.18)	(0.12)	(0.18)
Divorce, widowed	−0.33+	−0.23	−0.34+	−0.23	−0.34+	−0.25
	(0.18)	(0.29)	(0.18)	(0.29)	(0.18)	(0.29)
Region (Coastal region = 0)						
Central region	0.03	−0.11	0.04	−0.06	0.04	−0.06
	(0.09)	(0.15)	(0.09)	(0.15)	(0.09)	(0.15)
Western region	0.03	0.09	0.05	0.16	0.05	0.15
	(0.07)	(0.12)	(0.08)	(0.12)	(0.08)	(0.12)
Self-reported health (healthy = 0)						
Fair	0.13+	0.12	0.13+	0.14	0.13+	0.13
	(0.07)	(0.11)	(0.07)	(0.11)	(0.07)	(0.11)
Unhealthy	0.08	0.31+	0.09	0.28	0.09	0.31+
	(0.12)	(0.18)	(0.12)	(0.18)	(0.12)	(0.18)
Housing accumulation fund (No = 0)	0.35 ***	0.45 **	0.27 **	0.30 *	0.28 **	0.35 *
	(0.09)	(0.14)	(0.10)	(0.15)	(0.10)	(0.16)
Medical insurance (yes = 0)	0.03	−0.23+	0.06	−0.25+	0.06	−0.24+
	(0.08)	(0.13)	(0.08)	(0.13)	(0.08)	(0.13)
Job status (full-time job = 0)	−0.004	0.02	0.0001	−0.02	0.006	−0.003
	(0.10)	(0.16)	(0.10)	(0.16)	(0.10)	(0.16)
Workload/work intensity (independent = 0)						
Dependent on another’s decision partly	−0.12	−0.38 **	−0.15+	−0.39 **	−0.14+	−0.36 **
	(0.08)	(0.13)	(0.08)	(0.13)	(0.08)	(0.13)
Dependent on another’s decision entirely	0.05	−0.24 *	−0.002	−0.23+	0.01	−0.22+
	(0.07)	(0.12)	(0.08)	(0.12)	(0.08)	(0.12)
Working hour (40 or less = 0)						
41 to 50	−0.07	−0.13	−0.05	−0.08	−0.05	−0.09
	(0.09)	(0.14)	(0.09)	(0.14)	(0.09)	(0.14)
51 to 60	0.05	−0.20	0.07	−0.17	0.07	−0.17
	(0.09)	(0.14)	(0.09)	(0.15)	(0.09)	(0.15)
61 and more	−0.03	0.04	−0.01	0.09	−0.01	0.08
	(0.09)	(0.13)	(0.09)	(0.13)	(0.09)	(0.14)
Number of cigarettes smoked per day (No smoking = 0)						
Less than 10	−0.09	−0.40+	−0.09	−0.38+	−0.09	−0.39+
	(0.13)	(0.21)	(0.13)	(0.21)	(0.13)	(0.21)
10 to 19	−0.18	−0.57 **	−0.18	−0.56 **	−0.186+	−0.57 **
	(0.11)	(0.19)	(0.11)	(0.19)	(0.11)	(0.19)
20 and more	−0.12	−0.25+	−0.12	−0.24+	−0.12	−0.25+
	(0.09)	(0.14)	(0.09)	(0.14)	(0.09)	(0.14)
Frequency of drinking alcohol (No drink or once every few weeks = 0)						
1–2 times every week	0.12	0.36 **	0.12	0.37 **	0.12	0.37 **
	(0.09)	(0.14)	(0.09)	(0.14)	(0.09)	(0.14)
3–4 times every week	0.12	0.38+	0.12	0.38+	0.12	0.38+
	(0.14)	(0.21)	(0.14)	(0.21)	(0.14)	(0.21)
Drink every day	0.08	0.02	0.08	0.02	0.08	0.02
	(0.12)	(0.20)	(0.12)	(0.20)	(0.12)	(0.20)
Regular exercise (yes = 0)	−0.22 ***	−0.24 *	−0.22 ***	−0.25 *	−0.21 **	−0.24 *
	(0.06)	(0.10)	(0.07)	(0.10)	(0.07)	(0.10)
Frequency of social engagement	−0.002	0.01	0.001	0.02	−0.0001	0.01
	(0.01)	(0.02)	(0.01)	(0.02)	(0.01)	(0.02)
Familiarity with neighbor (Unfamiliar = 0)						
Fair	0.05	0.02	0.04	−0.01	0.04	−0.01
	(0.10)	(0.15)	(0.10)	(0.15)	(0.10)	(0.15)
Familiar	0.25 **	0.11	0.24 *	0.07	0.24 *	0.07
	(0.09)	(0.15)	(0.09)	(0.15)	(0.10)	(0.15)
_cons	−4.18 *	−6.21 *	−3.71 *	−6.00 *	−3.97 *	−6.14 *
	(1.67)	(2.81)	(1.66)	(2.81)	(1.67)	(2.82)
LR chi2	413.68		423.93		443.72	
Pseudo R^2^	0.038		0.039		0.040	

Note: significance level: *** *p* < 0.001, ** *p* < 0.01, * *p* < 0.05, + *p* <0.1; ^a^: the figure denotes unstandardized regression coefficient, ^b^: the figure in parentheses denotes the standard error.

## Data Availability

Publicly available datasets were analyzed in this study. This data can be found here: (“China Labor-force Dynamics Survey 2016” (CLDS 2016)) (cssdata@mail.sysu.edu.cn).

## References

[1] NCD Risk Factor Collaboration (2016). Trends in adult body-mass index in 200 countries from 1975 to 2014: A pooled analysis of 1698 population-based measurement studies with 19.2 million participants. Lancet.

[2] Ford N.D., Patel S.A., Narayan K.V. (2017). Obesity in low-and middle-income countries: Burden, drivers, and emerging chal-lenges. Annu. Rev. Publ. Health.

[3] Sharma A.M., Chetty V.T. (2005). Obesity, hypertension and insulin resistance. Acta Diabetol..

[4] Cloostermans L., Wendel-Vos W., Doornbos G., Howard B., Craig C.L., Kivimäki M., Tabak A.G., Jefferis B.J., Ron-kainen K., Brown W.J. (2015). Independent and combined effects of physical activity and body mass index on the develop-ment of Type 2 Diabetes—A meta-analysis of 9 prospective cohort studies. Int. J. Behav. Nutr. Phys. Act..

[5] Barquera S., Rivera J.A., Espinosa-Montero J., Safdie M., Campirano F., Monterrubio E.A. (2003). Energy and nutrient consumption in Mexicon women 12–49 years of age: Analysis of the National Nutrition Survey 1999. Salud Pubfica Mex..

[6] Hirko K.A., Lajous M., Ortiz-Panozo E., Lopez-Ridaura R., Christine P.J., Lȇ-Scherban F., Rice M.S., Barrien-tos-Gutierrez T. (2017). Socioeconomic position and markers of adiposity among female teachers in Mexico. J. Epidemiol. Community Health.

[7] Caballero B. (2007). The global epidemic of obesity: An overview. Epide. Rev..

[8] Dinsa G.D., Goryakin Y., Fumagalli E., Suhrcke M. (2012). Obesity and socioeconomic status in developing countries: A system-atic review. Obes. Rev..

[9] Godley J., McLaren L. (2010). Socioeconomic status and body mass index in Canada: Exploring measures and mechanisms. Can. Rev. Sociol..

[10] Yang T.-C., South S.J. (2018). Neighborhood effects on body mass: Temporal and spatial dimensions. Soc. Sci. Med..

[11] Jang H.-J., Oh H. (2021). Trends and inequalities in overall and abdominal obesity by sociodemographic factors in Korean adults, 1998–2018. Int. J. Environ. Res. Public Health.

[12] Jaacks L.M., Vandevijvere S., Pan A., McGowan C.J., Wallace C., Imamura F., Mozaffarian D., Swinburn B., Ezzati M. (2019). The obesity transition: Stages of the global epidemic. Lancet Diabetes Endocrinol..

[13] Yiengprugsawan V., Horta B., Motta J., Gigante D., Seubsman S.-A., Sleigh A. (2016). Body size dynamics in young adults: 8-year follow up of cohorts in Brazil and Thailand. Nutr. Diabetes.

[14] Newton S., Braithwaite D., Akinyemiju T.F. (2017). Socio-economic status over the life course and obesity: Systematic review and meta-analysis. PLoS ONE.

[15] Monteiro C.A., Moura E.C., Conde W.L., Popkin B.M. (2004). Socioeconomic status and obesity in adult populations of de-veloping countries: A review. Bull. World Health Organ..

[16] Fernald L.C. (2007). Socio-economic status and body mass index in low-income Mexican adults. Soc. Sci. Med..

[17] Jiwani S.S., Carrillo-Larco R.M., Hernández-Vásquez A., Barrientos-Gutiérrez T., Basto-Abreu A., Gutierrez L., Irazola V., Nieto-Martínez R., Nunes B.P., Parra D.C. (2019). The shift of obesity burden by socioeconomic status between 1998 and 2017 in Latin America and the Caribbean: A cross-sectional series study. Lancet Glob Health.

[18] Zhao P., Gu X., Qian D., Yan F. (2018). Socioeconomic disparities in abdominal obesity over the life course in China. Int. J. Equity Health.

[19] Hou X., Jia W., Bao Y., Lu H., Jiang S., Zuo Y., Gu H., Xiang K. (2008). Risk factors for overweight and obesity, and changes in body mass index of Chinese adults in Shanghai. BMC Public Health.

[20] Wang Y., Mi J., Shan X.-Y., Wang Q.J., Ge K.-Y. (2007). Is China facing an obesity epidemic and the consequences? The trends in obesity and chronic disease in china. Int. J. Obes..

[21] Cai L., He J., Song Y., Zhao K., Cui W. (2013). Association of obesity with socioeconomic factors and obesity-related chronic diseases in rural southwest China. Public Health.

[22] Wang H., Wang J., Liu M.-M., Wang D., Liu Y.-Q., Zhao Y., Huang M.-M., Liu Y., Sun J., Dong G.-H. (2012). Epidemiology of general obesity, abdominal obesity and related risk factors in urban adults from 33 communities of Northeast China: The CHPSNE study. BMC Public Health.

[23] Lao X.Q., Ma W., Chung R.Y.-N., Zhang Y., Xu Y., Xu X., Nie S., Cai Q., Xia L., Su X. (2015). The diminishing socioeconomic disparity in obesity in a Chinese population with rapid economic development: Analysis of serial coss-sectional health survey data 2002–2010. BMC Public Health.

[24] Fu Q., George L.K. (2015). Socioeconomic determinants of childhood overweight and obesity in China: The long arm of institutional power. Sociol. Health Illn..

[25] Scott W., Richard D. (2013). Institutions and Organizations: Ideas, Interests, and Identities.

[26] Wu X. (2013). Redrawing the boundaries: Work units and social stratification in Urban China. Chin. Sociol. Rev..

[27] Xie Y., Wu X. (2008). Danwei profitability and earnings inequality in Urban China. China Q..

[28] Mulatu M.S., Schooler C. (2002). Causal connections between socio-economic status and health: Reciprocal effects and mediating mechanisms. J. Health Soc. Behav..

[29] Cockerham W.C., Cockerham W.C. (2013). Bourdieu and an update of health lifestyle theory. Medical Sociology on the Move.

[30] Christensena V.T., Carpiano R.M. (2014). Social class differences in BMI among Danish women: Applying Cockerham’s health lifestyles approach and Bourdieu’s theory of lifestyle. Soc. Sci. Med..

[31] Sobal J., Stunkard A. (1989). Socioeconomic status and obesity: A review of the literature. Psychol. Bull..

[32] Zienczuk N., Egeland G. (2012). Association between socioeconomic status and overweight and obesity among Inuit adults: International Polar Year Inuit Health Survey, 2007–2008. Int. J. Circumpolar Health.

[33] Lartey S.T., Magnussen C.G., Si L., de Graaff B., Biritwum R.B., Mensah G., Yawson A., Minicuci N., Kowal P., Boateng G.O. (2019). The role of intergenerational educational mobility and household wealth in adult obesity: Evidence from Wave 2 of the World Health Organization’s Study on global AGEing and adult health. PLoS ONE.

[34] Espositoa L., Villasenor A., Rodriguez E.C., Millet C. (2020). The economic gradient of obesity in Mexico: Independent predic-tive roles of absolute and relative wealth by gender. Soc. Sci. Med..

[35] Devaux M., Sassi F., Church J., Cecchini M., Borgonovi F. (2011). Exploring the relationship between education and obesity. OECD J. Eco. Stud..

[36] Block J.P., He Y., Zaslavsky A.M., Ding L., Ayanian J.Z. (2009). Psychosocial stress and change in weight among us adults. Am. J. Epidemiol..

[37] Ostry A.S., Radi S., Louie A.M., LaMontagne A.D. (2006). Psychosocial and other working conditions in relation to bodymass index in a representative sample of Australian workers. BMC Public Health.

[38] Fernandez I.D., Su H., Winters P.C., Liang H. (2010). Association of workplace chronic and acute stressors with employee weight status: Data from worksites in turmoil. J. Occup. Environ. Med..

[39] Kishi T., Elmquist J.K. (2005). Body weight is regulated by the brain: A link between feeding and emotion. Mol. Psychiatry.

[40] Kivimäki M., Head J., Ferrie J.E., Shipley M.J., Brunner E., Vahtera J., Marmot M.G. (2006). Work stress, weight gain and weight loss: Evidence for bidirectional effects of job strain on body mass index in the White hall II study. Int. J. Obes..

[41] Kouvonen A., Kivimaki M., Vaananen A., Heponiemi T., Elovainio M., Ala-Mursula L., Virtanen M., Pentti J., Linna A., Vahtera J. (2007). Job strain and adverse health behaviors: The Finnish public sector study. J. Occup Environ. Med..

[42] Kouvonen A., Kivimaki M., Cox S.J., Cox T., Vahtera J. (2005). Relationship between work stress and body mass index among 45,810 female and male employees. Psych. Med..

[43] Wardle J., Steptoe A., Oliver G., Lipsey Z. (2000). Stress, dietary restraint and food intake. J. Psych. Res..

[44] Li S., Chen Y., He G. (2018). Laugh and grow fat: Happiness affects body mass index among Urban Chinese adults. Soc. Sci. Med..

[45] Brunner E.J., Chandola T., Marmot M.G. (2007). Prospective effect of job strain on general and central obesity in the White-hall II study. Am. J. Epidemiol..

[46] Hannerz H., Albertsen K., Nielsen M.L., Tuchsen F., Burr H. (2004). Occupational factors and 5-year weight change among men in a Danish national cohort. Health Psychol..

[47] Liu X. (2005). Understanding the mechanism of social stratification in contemporary China. Sociol. Stud..

[48] Cao Y., Rubin B.A. (2014). Market transition and the deinstitutionalization of standard work hours in Post-Socialist China. Ind. Labor Relat. Rev..

[49] Wu Y., Huang P., Huang C. (2015). Family, institutional patronage and work-family conflict: Women’s employment status and subjective well-being in Urban China. Sociol. Stud..

[50] Bian Y. (2018). The prevalence and the increasing significance of Guanxi. China Q..

[51] Hwang K. (1987). Face and favor: The Chinese power game. Am. J. Soc..

[52] Wang J., Zhou Y., Liu S. (2017). China labor-force dynamics survey: Design and practice. Chin. Sociol. Dialogue.

[53] Levasseur P. (2015). Causal effects of socioeconomic status on central adiposity risks: Evidence using panel data from urban Mexico. Soc. Sci. Med..

[54] Xie Y., Lai Q., Wu X. (2009). Danwei and social inequality in contemporary urban China. Res. Soc. Work.

[55] Pei L., Cheng Y., Kang Y., Yuan S., Yan H. (2015). Association of obesity with socioeconomic status among adults of ages 18 to 80 years in rural Northwest China. BMC Public Health.

[56] Pengpid S., Peltzer K. (2021). Overweight and Obesity among Adults in Iraq: Prevalence and correlates from a national survey in 2015. Int. J. Environ. Res. Public Health.

[57] Sengupta A., Angeli F., Syamala T.S., Dagnelie P.C., Van Schayck C.P. (2015). Overweight and obesity prevalence among Indian women by place of residence and socio-economic status: Contrasting patterns from ‘underweight states’ and ‘overweight states’ of India. Soc. Sci. Med..

[58] Quezada A.D., Lozada-Tequeanes A.L. (2015). Time trends and gender differences in asso-ciations between socioeconomic status indicators and overweight-obesity in Mexico (2006–2012). BMC Public Health.

[59] Bonnefond C., Clement M. (2014). Social class and body weight among Chinese urban adults: The role of the middle classes in the nutrition transition. Soc. Sci. Med..

[60] Juwara A., Huang N., Chien L.Y., Chen H.J. (2016). Stunting and weight statuses of adolescents differ between public and pri-vate schools in urban Gambia. Int. J. Public Health.

[61] Drewnowski A., Specter S.E. (2004). Poverty and obesity: The role of energy density and energy costs. Am. J. Clin. Nutr..

[62] House J.S. (2002). Understanding social factors and inequalities in health: 20th Century Progress and 21st Century Prospects. J. Health Soc. Behav..

[63] Böckerman P., Viinikainen J., Pulkki-Råback L., Hakulinen C., Pitkänen N., Lehtimäki T., Pehkonen J., Raitakari O.T. (2017). Does higher education protect against obesity? Evidence using Mendelian randomization. Pre. Med..

[64] Zhang H., Xu H., Song F., Xu W., Pallard-Borg S., Qi X. (2017). Relation of socioeconomic status to overweight and obesity: A large population-based study of Chinese adults. Ann. Hum. Biol..

[65] Kim Y.-J. (2016). The long-run effect of education on obesity in the US. Econ. Hum. Biol..

[66] Mayén A.-L., Marques-Vidal P., Paccaud F., Bovet P., Stringhini S. (2014). Socioeconomic determinants of dietary patterns in low-and middle-income countries: A systematic review. Am. J. Clin. Nutr..

[67] Luo Y., Parish W.L., Laumann E.O. (2005). A population-based study of body image concerns among urban Chinese adults. Body Image.

[68] Bray D. (2005). Social Space and Governance in Urban China: The Danwei System from Origins to Reform.

[69] Bongers P., Jansen A., Havermans R., Roefs A., Nederkoorn C. (2013). Happy eating: The underestimated role of overeating in a positive mood. Appetite.

